# Promoting Sports Engagement during the COVID-19 Pandemic via Virtual Reality Games

**DOI:** 10.1155/2022/4824152

**Published:** 2022-01-25

**Authors:** Hana Hanifah, Yuko Ito, Daryl Patrick Gamboa Yao, Natsuka Suyama, Kaoru Inoue

**Affiliations:** ^1^Department of Occupational Therapy, Graduate School of Human Health Sciences, Tokyo Metropolitan University, Japan; ^2^Department of Disability and Human Development, College of Allied Health Sciences, The University of Illinois at Chicago, USA

## Abstract

**Objective:**

To examine sports engagement and health changes of young adults when utilizing a VR sports game and investigate the relationship between sports engagement and health.

**Method:**

This study used a single-group design with 20 participants, aged 19–29 years, with no preexisting health conditions. The VR game “Sports Scramble” was used thrice within a span of one week. Outcomes sought include sports engagement and health, measured through the Sports Engagement Scale (SES) and Short Form 36 (SF-36), respectively.

**Results:**

A significant difference (*p* < 0.001) was found between the pre-posttest scores of the SES. Moreover, a positive trend was observed in terms of health with a significant difference (*p* < 0.05) between pre-posttest scores of the SF-36's vitality dimension. There were positive correlations among the dedication and vigor subscales of the SES with the dimensions of health.

**Conclusion:**

This study showed the potential of VR sports games in positively influencing sports engagement and health among participants with the vigor and dedication positively influencing health. Future studies may involve the exploration of the effectiveness of VR to promote engagement and health through a randomized controlled trial with a longer timeframe and across various populations.

## 1. Introduction

Sports engagement is a bond between an individual and sport-related activities that is marked by positive cognition, attitudes, and emotion bringing about a feeling of immersion [1] leading to positive health and well-being not just physically but also emotionally and mentally [2]. Despite the health advantages of engaging in sport, a survey done by England's Department of Culture, Media, and Sport (2012) found a negative correlation between the number of individuals who engage in sports and age. Additionally, the level of physical activity performed drops below the recommended level as they reach young adulthood [3]. Because physical activities appear to be uninteresting and unenjoyable due to the repetitive movements embedded within most activities [4], young adults may tend to prefer and find greater intrinsic meaning to more sedentary activities such as watching television and playing videogames.

This puts young adults at higher risk to poor health especially as they are already susceptible to unintentional health concerns such as unhealthy lifestyles, illegal substances, obesity, and physical inactivity [5]. In 2002, National Co-Morbidity Survey revealed that 75% of the total diagnosed mental issues start after age 24, with 52.4% of individuals aged 18–29 experiencing mental health concerns (Kessler, et. al., [6]). The most common diagnoses in this age group are depression (15.4%) and alcohol abuse (14.3%) [5].

Additionally, the impact of the COVID-19 pandemic is massive in perpetuating the engagement in these sedentary activities [7] and health concerns among young adults [8]. The strict social distancing and cordon sanitaire influenced many aspects of one's life. The closure of public places, fitness centers, gymnasiums, pools, parks, universities, and playgrounds has successfully hampered routine fitness activities resulting in various fitness and health concerns [9] and isolating people from their social circle. Additionally, several surveys found a decline in physical activities and increased sedentary activities, especially among young adults, during the COVID-19 pandemic [10–12].

Among many sedentary activities, the demand for consuming video games has grown extensively during the pandemic [13]. During the peak time of COVID-19 lockdowns, 82% of global consumers have played or watched video games [11]. Nevertheless, playing video games is not without merits. Recent studies found that video games and other related technologies can play a role in continuing social connections, providing psychological healing and relief, and maintaining well-being during global quarantines as a result of COVID-19 [11].

Nowadays, playing video games can potentially serve as a medium for exercise and physical activities as well. Numerous active video games using virtual reality (VR) technology played in different consoles such as the Oculus®, Wii-Fit®, Xbox360 Kinect®, and PlayStation-Move® are gaining popularity. In addition to creating a fun, upbeat, and entertaining atmosphere [14], active video games generally result in higher energy expenditure [15].

The primary purpose of VR is to create a simulated environment to provide an experience as near as one's real environment [16]. VR experience could be appraised by looking at the presence and realism offered by a device wherein implementing a higher degree of presence of virtual stimuli would have a greater effect on user behavior [17]. While the current VR technology needs improvement in the accuracy, precision, and latency of their tracking system [18], VR-mediated sports are found to be effective in improving athletic performance, particularly in soccer goalkeeping [19], rowing [20], surfing [21], and marksmanship [22], among others. Moreover, utilizing the VR technology in healthcare practice has been explored on sequelae of conditions across the lifespan [23]. The most popular use of VR technology in healthcare is assessing and providing treatment for individuals with mental health disorders [24].

VR is a new technology that has also been used in occupational therapy (OT) practice [25, 26]. OT plays a vital role in promoting health and well-being through engagement in occupations [27]. Harnessing VR technology in OT can be beneficial to induce enjoyment and engagement in meaningful activities for numerous populations as it impacts multiple client factors, motivation, and internal locus of control and provides avenues for noncontact engagement in activities [23, 26, 28].

Integrating VR as a means for sports engagement can be beneficial for improving health outcomes in young adults [25]. However, few studies explored the potential of VR-mediated sports engagement amidst the COVID-19 pandemic. Therefore, this study mainly is aimed at (1) examining the sports engagement and health when utilizing a commercial VR sports game in young adults and (2) investigating the relationship between sports engagement and health after VR sports games. Understanding the benefits of VR sports games may assist in developing future interventions for the health promotion of various populations.

## 2. Methods

### 2.1. Study Design, Setting, and Sample Size

This is a pre-experimental study conceptualized to explore a novel idea of utilizing VR to promote engagement and health during the COVID-19 pandemic. A pre-experimental design is often used before conducting the true experiment to examine if the intervention can affect a potential small group [29]. To acquire data, this study was conducted using a one-group pretest-posttest design. The study was conducted within the premise of Tokyo Metropolitan University, Tokyo, Japan, wherein a spacious, clutter-free, well-lit, well-ventilated room was specially allocated for data collection and intervention implementation.

Considering the feasibility, timeframe, and limitations of the current situation, wherein regulations for limited contact were enforced and the threat posed by COVID-19 was great, the sample size was determined in adherence to the suggestion by Birkett and Day [30] of acquiring a minimum sample size of 20. Thus, a sample size of 20 is targeted for this study.

### 2.2. Participants

Participants were selected through a nonrandomized, purposive sampling. Inclusion criteria were (1) young adults aged 19 to 29 years, (2) without any pre-existing health condition, (3) can understand English, (4) are familiar with video games, and (5) engaged in leisurely physical activities before the outbreak of COVID-19. Individuals who engaged in professional athletic competitions within the past five years were excluded from the study.

Participants were primarily recruited through recruitment posters advertised in the university bulletin to inform potential participants to volunteer for this study. Additionally, snowball recruitment occurred wherein earlier participants encouraged their peers to volunteer for the study. When an individual is interested in volunteering, the individual contacts the primary investigator and schedules an in-person meeting to discuss the informed consent process and, if possible, the first session of the intervention.

### 2.3. Intervention

**Figure 1:**
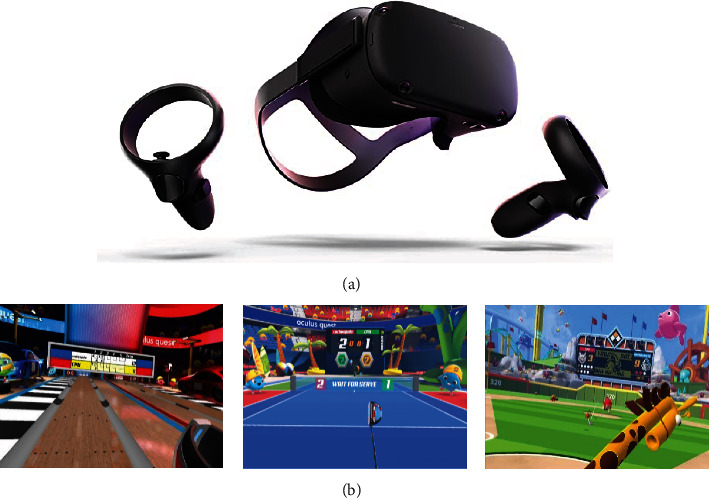
Tools and scenario used in intervention: (a) Oculus Quest with a controller and (b) three types of sport scenarios in Sport Scramble. Bowling, tennis, and baseball (left to right).

A VR device with two controllers named “Oculus Quest” was utilized as the tool for the intervention (Figure 1). A commercial sport-based game called “*Sports Scramble*,” which has three different sports, namely, tennis, baseball, and bowling was utilized in this study. These sports were chosen in this study as they are popular among younger adults [31].

The entirety of data collection and intervention implementation took place from March to April 2021. The VR game was mirrored on a separate tablet to allow the primary investigator to monitor and observe the occurrences within the virtual world. The playing field was preset with a diameter of 2 meters. The VR was placed at the center of the room while the primary investigator observes from the sideline outside the predetermined playing field. Participants were recommended to wear comfortable clothes and shoes to allow for unrestricted movement.

The intervention took place in three distinct sessions lasting for a total of approximately 45–60 minutes per session, with sessions held one to two days apart, depending on the participant's availability. Each participant was allocated a maximum of one week to complete the protocol. The primary investigator facilitated data collection and intervention while one coauthor provided logistic support through setting up the room and sanitizing the equipment. The intervention was conducted in adherence to preventive protocols of infectious disease including maintaining proper air circulation, hand hygiene, device hygiene, limited room capacity with the enforcement of a strict scheduling system, and wearing of face masks.

The first session consists of a pretest wherein baseline information, including the demographic information for gender, age, sports activity profile, and the familiarity of VR games, was collected. Sports engagement and health prior to the intervention were also obtained. After collecting baseline data, the participant was asked to select one of the three available sports based on their interests. The order and the kind of task within the intervention were similar across all three sports. Regardless of the sport selected, the data obtained were collectively considered as one group. The participant was then oriented with basic functions related to the VR device, the game, and things to expect upon entering the realm of VR. Participants had a 30-minute training session to practice their virtual hands, adjust to the virtual environment, and familiarize themselves with the rules of the game. Verbal prompts and cues were provided appropriately. The first postintervention assessment for sports engagement was done afterwards.

**Figure 2:**
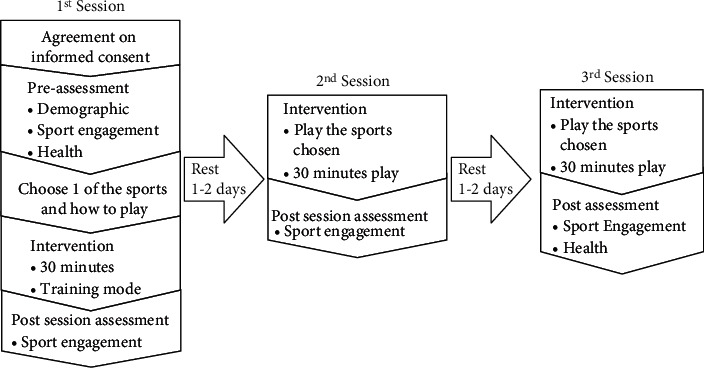
Intervention flowchart.

One to two days of rest were provided between sessions to accommodate muscle soreness that may be felt postphysical activity. Within the second and third sessions, participants were given a chance to explore the features of the sport they had initially chosen, which can be either a normal match with a just-right difficulty or challenges embedded in the game. The just-right difficulty was on the primary investigator's prerogative based on the participant's performance from the previous session. The participants were encouraged to ask any game-related questions. Participants were observed and visuals from the VR game were available to the researcher in real time to provide accurate instructions. Each intervention was delivered for 30 minutes or when the participants requested to stop due to cybersickness. At the end of every session, an assessment for sports engagement was performed. At the end of the third session, data on health was collected.  Figure 2 provides a visual overview of the intervention protocol.

### 2.4. Outcomes

#### 2.4.1. Sport Engagement

Sport engagement refers to their sport-related psychological processes and sports performance assessed using the Sport Engagement Scale (SES) [32]. The participant's SES was obtained at the pretest and the end of every session. The SES is a self-report tool that consists of 15 statements rated on a 7-point Likert-type scale. Three main points assessed in this instrument are vigor (willingness to invest in the activity), dedication (high level of involvement), and absorption (level of immersion). The research by Guillen and Martinez-Alvarado [32] reported that the SES had a Cronbach's *α* of 0.75 for all subscales and *α* = 0.90 for the total scale thereby establishing its reliability and fulfills the validity of the construct with *r* values oscillating between 0.49 and 0.83 when compared to sport burnout factors indicating a strong correlation.

#### 2.4.2. Health

Health was measured during preintervention and at the end of the intervention protocol using the Short Form 36 (SF-36). SF-36 is a 36-item self-report questionnaire that was used to measure health according to eight dimensions, namely, physical functioning, social functioning, role limitations due to physical problems, role limitations due to emotional problems, mental health, vitality, bodily pain, and general health (Jenkinson, [33]). Raw scores were scaled into a score between zero and a hundred for each dimension. Higher scores indicated better health. These dimensions make up either the physical component scales (PCS) or the mental component scales (MCS) [34]. Moreover, vitality and general health dimensions were analyzed separately to present well-being and personal health evaluation as these dimensions can measure a wider range of negative and positive sides of the health state as mid-range scores reported appear to be the best condition [34]. Jenkinson et al. [35], measured the internal consistency of the variables in the SF-36 among the working-age group which resulted in a Cronbach's *α* of 0.5 or above, which is usually deemed acceptable. They also assessed and determined the criterion validity of the tool by obtaining a statistically significant trend when comparing SF-36 scores with a global health question.

### 2.5. Ethics

The ethics committee (blinded for review) reviewed and approved the protocol of this study (approval number: 20078). The information collected and procedure done were in adherence to the stipulations of the Declaration of Helsinki and the policies concerning human participants stipulated by the institution that has authority over the authors. Written informed consents were obtained before the intervention. Prior to affixing their signature, participants were thoroughly briefed about the content of the informed consent including an overview of the research, compensation (a gift card worth ¥3,000, which is approximately $26), possible harm from the intervention (muscle soreness and cybersickness), and their right to withdraw at any time without incurring any penalty, among others, and were given the chance to ask for clarifications regarding the intervention.

### 2.6. Statistical Analysis

All the statistical analysis was performed using JASP v0.14 [36]. Outliers were assessed graphically through a boxplot and underwent 90% winsorization to minimize its influence during analysis [37]. We acknowledge, however, that these outlier data on our sample size are reflective of our participant's reality during data collection. Appropriate descriptive statistical data of the variables, such as frequency distributions, median and interquartile ranges, and mean and standard variation, were generated for demographic information, SES, and SF-36, as appropriate. Tests of significance were based on an alpha level of 0.05 and the confidence level was set at 95%.

One-way repeated measures analysis of variance (RM-ANOVA) with four levels was used to analyze the SES score. If the test of sphericity, indicated by Mauchly's *W*, was violated thereby increasing the risk of a Type I error, *F*-statistic was corrected using the Greenhouse–Geiser adjustment. Only vigor and dedication were found to have statistical differences and underwent correction. Whenever significant differences were found, a post hoc comparison was performed using the Bonferroni method to uncover specific differences between sessions of intervention. Effect sizes were calculated using an adaptation of omega (*ω*^2^) within the subject design.

SF-36, on the other hand, was first analyzed using the Shapiro-Wilk test for normality. As vitality and general health revealed statistical differences (*p* < 0.05), Wilcoxon signed-rank test was used to examine differences between preintervention and postintervention. The paired *t*-test was then used to compare preintervention and postintervention of MCS and PCS. Effect sizes were calculated using rank-biserial correlation (*r*_*B*_) for vigor and general health and Cohen's *d* for MCS and PCS.

Pearson's correlation test was performed to investigate whether there is statistical evidence for a linear relationship between SES and SF-36 variables postintervention. When variables were deemed significantly and strongly correlated, the coefficient of determination denoted by *R* squared (*R*^2^) is used to represent the proportion of the variance for the SF-36 dimension that is explained by the SES subscale.

## 3. Results

### 3.1. Demographic and Participant Sport Profile

Twenty participants volunteered and completed the study. The demographic characteristics of the participants are summarized in Table 1. All participants are university students pursuing either their bachelor's or postgraduate studies. The sex ratio was approximately equal. Most participants are right handed. In addition, 45% of participants showed a diminished frequency of performing sports per week since the outbreak of COVID-19, while 30% stated that their frequency of doing sports per week remained unchanged. Among participants, 45% are familiar with VR gaming.

### 3.2. Sport Engagement

**Figure 3:**
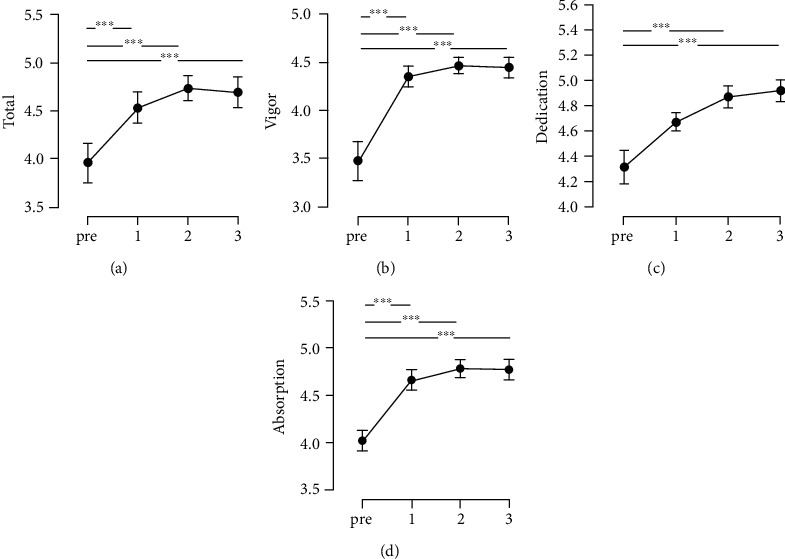
Results of the Sport Engagement Scale total score and each factor score through each intervention. *y*-axis: raw score of SES; *x*-axis: intervention timeline. Error bars represent standard error (SE). (a) Total score of SES; (b) vigor; (c) dedication; (d) absorption. ^∗∗∗^*pbonf* < 0.001.

The level of sports engagement significantly increased from the preintervention to postintervention (Figure 3). A significant increase in the level of engagement was found on the means score postintervention in contrast to the preintervention in all factors (Figure 3). Total scores of the sports engagement scale showed significant differences between means of the differences across sessions *F*(3, 57) = 20.37, *p* < 0.001 (CI = [0.43, 1.04]) with a large effect (*ω*^2^ = 0.21). Furthermore, significant differences were also found between the mean of differences in each subscale of the scale; vigor *F*(1.71, 32.46) = 12.48, *p* < 0.001 (CI = [0.45, 1.48]) with large effect (*ω*^2^ = 0.22); dedication *F*(2.02, 38.37) = 8.06, *p* = 0.001 (CI = [0.23, 0.99]) with medium effect (*ω*^2^ = 0.11); and absorption *F*(3, 57) = 12.24, *p* < 0.001 (CI = [0.35, 1.16]) with medium effect (*ω*^2^ = 0.12). Post hoc analyses revealed significant differences in every subsequent session when compared to baseline data as shown in Figure 3. After the first session, a drastic increase in the engagement level was observed as well as a gradual increase following the subsequent sessions.

### 3.3. Health

**Figure 4:**
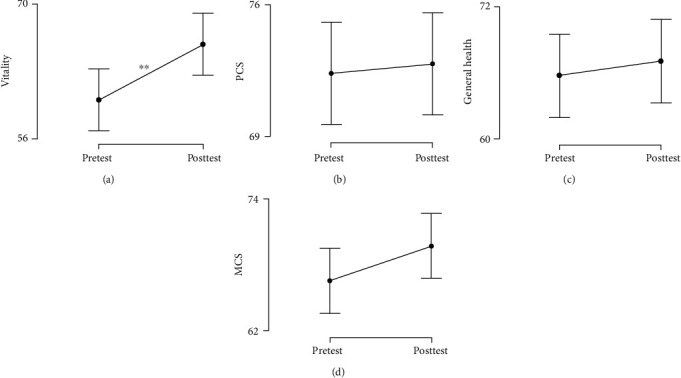
Comparison of SF-36 dimensions and scales between pretest and posttest. *y*-axis: score of SF-36; *x*-axis: intervention timeline. Error bars represent standard error (SE); dot represents the mean. (a) Vitality; (b) physical component scale; (c) general health; (d) mental component scale. ^∗∗^*p* < 0.01.

Scores of SF-36 increased between pre-posttest for all scales (Figure 4). However, only the vitality dimension exhibited a statistically significant increase between pretest (Mdn = 65, IQR = 25) and posttest (Mdn = 70, IQR = 17.5) scores (*W* = 106, *p* < 0.01, CI = [0.41, 0.92]) with a large effect size (*r*_*B*_ = 0.77).

### 3.4. Relationship between Sport Engagement and Health

**Figure 5:**
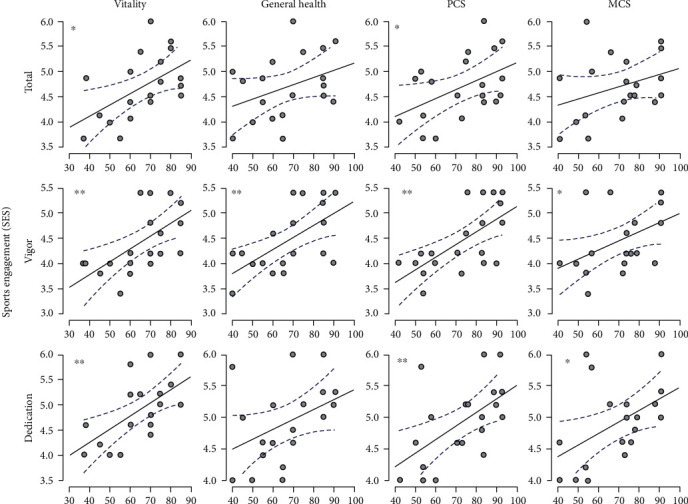
Pearson's correlation scatterplot between subscales of SES and SF-36. *x*-axis indicates SF-36 score; *y*-axis shows SES score. Dashed curves indicate lower and upper bounds of confidence interval. ^∗^*p* < 0.05; ^∗∗^*p* < 0.01.

After engaging in VR sports, the Pearson's correlation showed positive significant correlations between several SES subscales and the SF-36 components (Figure 5). No correlation was found between absorption and components of SF-36. The total sports engagement score showed a significant positive and moderate association with PCS (*r* = 0.47, *p* < 0.05, CI = [0.03, 0.75]) and vitality (*r* = 0.53, *p* < 0.05, CI = [0.11, 0.79]). A significant, strong, and positive correlation between vitality and dedication in sport (*r* = 0.623, *p* < 0.01, CI = [0.25, 0.84]) was observed with dedication accounting for 38.8% of the variance in vitality score. Moreover, dedication exhibited moderately positive correlation between PCS (*r* = 0.57, *p* < 0.01, CI = [0.17, 0.81], *R*^2^ = 32.5%) and MCS (*r* = 0.49, *p* < 0.05, CI = [0.06, 0.77], *R*^2^ = 24%). PCS and vigor in sport exhibited significant, strong, and positive correlation (*r* = 0.65, *p* < 0.01, CI = [0.29, 0.85]) with vigor accounting for 42.3% of the variance in PCS score. Vigor has also exhibited strong and positive association with general health (*r* = 0.61, *p* < 0.01, CI = [0.23, 0.83], *R*^2^ = 37.2%) and vitality (*r* = 0.61, *p* < 0.01, CI = [0.22, 0.83], *R*^2^ = 37.2%) and moderate and positive association with MCS (*r* = 0.48, *p* < 0.05, CI = [0.05, 0.76], *R*^2^ = 23%).

## 4. Discussion

Our study is aimed at examining sports engagement and health when utilizing a commercial VR game in young adults and the relationship between sports engagement and health. Our findings demonstrated a high level of engagement in VR-mediated sports games, suggesting that VR sports can be a reliable alternative for sports activity. Moreover, our findings revealed that despite not seeing a correlation between the total scores of SES and SF-36, a significant positive and moderate association between several components of SES and SF-36 exists.

The overall score of the SES, including its components, significantly improved after three sessions of engagement in VR sports. The findings support the claims made by O'Donovan and associates [3] that active video games can encourage sedentary adults to be more active and engaged. While they caution against replacing traditional physical activities with videogames [3], we argue that it is a welcome alternative, especially during the COVID-19 pandemic. This is in alignment with Yoo et al. [38] wherein they welcome the substitution of VR games to traditional exercises for people involved in sedentary jobs as it is still a useful source of physical exertion and activity. Additionally, a VR study in respect to leisure sport reported that VR sports game facilitates leisure satisfaction and sustainable engagement [39].

The effect of sport engagement for young adults can be directly associated with physical activities with long-term secondary effects. While our study did not yield a significant difference in terms of health, this can be attributed to the high pretest score and significantly short intervention duration. Nonetheless, the increasing trend noticed after the intervention indicates that health improvement can still be present and VR games may be beneficial towards a healthier lifestyle. Sports is an essential occupation with benefits for health and well-being [40]. As Malm et al. [41] argued, physical activities and exercise lead to an active life, thereby improving young people's physical and mental health potentially declining morbidity and mortality.

A positive correlation between sports engagement and health-related factors was noted after performing VR sports games. Based on these findings, each component of health has a correlation with sports engagement except for *absorption*. Interestingly, *dedication* and *vigor* became essential factors associated with improving health while performing the VR sport. These factors were said to increase the performance level without being related to skill and ability [42]. Our finding determined that *vigor* is associated with vitality. *Vigor* promotes various resources related to physical activity, and vitality is more related to mental energy [43]. *Vitality* implies a sense of enthusiasm, liveliness, and energy available for oneself [44, 45]. The increased feeling of vigor from performing physical activities can decrease persistent fatigue [46].

Our finding revealed that *dedication* is correlated with the mental component, suggesting that engaging in VR sports games could contribute to mental health. A study with adolescents in Canada also stated that dedication in sport may lower depression symptoms and protect against poor mental health in adulthood [47]. Engagement in meaningful activities, in this case performing sports in VR games, can distract from stressful situations to promote the sense of doing, being, and becoming as to become the person one aspires to be [48].

Collectively, our findings demonstrated VR-mediated sports can be a fun, engaging, potentially healthy way to disrupt sedentary lifestyles perpetuated by the COVID-19 pandemic. Sports engagement might increase life satisfaction, physical and psychological health, and quality of life [49]. OT practitioners play a role in promoting health and well-being by guiding people to engage in physical activity in their routine [50]. The health and safety protocols during the COVID-19 pandemic period limited the chance to engage in various occupations, including sports. While sports engagement is not having enough attention among the scopes of OT [51], promoting health and well-being through engaging in VR sport games is an excellent opportunity for OT practice.

### 4.1. Limitation and Recommendation

This present study has several limitations. Firstly, being a one-group pilot study by design, there is a small sample size with a possibility of a Hawthorne effect. Thus, the results of this study need to be explored further through conducting a randomized controlled trial with a possibly longer timeframe before being generalizable. Nevertheless, this study was able to elucidate the possibilities of utilizing VR sports to promote engagement and health despite the social limitations and disruptions brought about by the COVID-19 pandemic. Secondly, there might be bias in terms of representation as only university students participated in this study. We recommend recruiting participants from a wider population. Lastly, while sports engagement was observed, the accuracy of movement to actual sports needs to be explored. In future studies, we recommend investigating the physical reaction and movement while playing VR sports games and comparing them to standard movement on the respective sport.

## 5. Conclusions

In summary, this study showed the potential of VR sports games to promote sports engagement and health. Dedication and vigor are the major factors needed to engage in VR sports games. The health aspect greatly influenced by VR sports engagement is vitality. With the existing restrictions due to the COVID-19 pandemic, virtually engaging in VR sports may potentially serve as a means to continue engaging in meaningful outdoor activities, including sports, thereby promoting an active lifestyle and health.

## Figures and Tables

**Table 1:** Participant characteristics (*n* = 20).

	Mean (SD)	Frequency (%)
Age	26.65 (3.46)	
*Gender*		
Male		11 (55%)
Female		9 (45%)
*Change in frequency of sport activity during COVID-19*		
Increased frequency per week		5 (20%)
Diminished frequency per week		6 (30%)
No changes		9 (45%)
*Sport selected for the intervention*		
Tennis		10 (50%)
Baseball		8 (40%)
Bowling		2 (10%)
*Familiarity with VR*		
No idea		4 (20%)
Heard about it		1 (5%)
Know about it		9 (45%)
Experienced it		6 (30%)

SD: standard deviation.

## Data Availability

The data used to support the findings of this study are included within the article. However, for more information, requests for access to these data should be made to Hana Hanifah (hana.ot13@gmail.com).
